# Genome Sequence of Avirulent *Riemerella anatipestifer* Strain RA-JLLY

**DOI:** 10.1128/genomeA.00895-15

**Published:** 2015-09-24

**Authors:** Tengfei Zhang, Rongrong Zhang, Qingping Luo, Guoyuan Wen, Diyun Ai, Honglin Wang, Ling Luo, Hongcai Wang, Huabin Shao

**Affiliations:** Hubei Key Laboratory of Animal Embryo and Molecular Breeding, Institute of Animal Husbandry and Veterinary, Hubei Academy of Agricultural Sciences, Wuhan, China

## Abstract

*Riemerella anatipestifer* is an important bacterial pathogen associated with epizootic infections in waterfowl and various other birds. *Riemerella anatipestifer* strain RA-JLLY is an avirulent strain, isolated from the brain of an old duck in Hubei province, China. Here, we report the genome sequence of this species.

## GENOME ANNOUNCEMENT

*Riemerella anatipestifer* (RA) is a Gram-negative, nonmotile, and non-spore-forming rod-shaped bacterium for which 21 serotypes have been identified (1, 2). RA causes the contagious septicemic disease in waterfowl and various other birds resulting in an enormous loss to poultry production (3). However, only a few virulence factors of RA have been identified (4–6), and not much is known about the pathogenesis. *R. anatipestifer* RA-JLLY, a strain isolated from the brain of an old duck in China, has been proved to be avirulent in our test. Here, the genome of RA-JLLY was sequenced. We think the sequence of this interesting strain will help us to further understand the pathogenesis of *R. anatipestifer* by comparison with the sequenced virulent strains (7, 8).

Two libraries containing 180-bp fragments and 500-bp fragments were constructed, respectively. High-throughput sequencing was performed using Illumina HiSeq2000 systems in Beijing, China. Finally, the sequence reads were assembled into 16 scaffolds (*N*_50_ length, 305,986 bp) using SOAP*denovo*. Protein-coding genes were predicted using Prodigal (9). Tandem repeat were detected by RepeatMasker. tRNA and rRNA genes were identified by tRNAscan and RNAmmer, respectively (10, 11). Functional annotation was performed by searching against protein databases, including COG and KEGG (12, 13).

The RA-JLLY chromosome is 2,189,463 bp in length, with an average G + C content of 35.1%. There are 2,019 putative open reading frames with an average length of 985 bp, and the coding percentage is 90.81%. It also contains 40 tRNA genes and 30 rRNA genes. 1,316 genes could be assigned to COG families, and 754 genes were designated to a KEGG pathway. A total of 4,199 bp containing 61 repeat sequences were detected in the genome. Similar to another avirulent *R. anatipestifer*, RA-SG, two credible clustered regularly interspaced short palindromic repeats (CRISPRs) and two possible CRISPRs were found (14). In addition, there is an 89-bp prophage sequence. Urease, which can convert urea into ammonia and carbamic acid, has been regarded as a virulence factor in some pathogens (15, 16). However, urease gene cluster, including *ureABCDEF* which has been reported in other *R. anatipestifer* spp., was not identified in RA-JLLY.

Overall, the genome sequence of avirulent *R. anatipestifer* RA-JLLY will provide the genetic background for understanding the pathogenicity mechanisms of this pathogen using comparative genomics.

### Nucleotide sequence accession numbers.

This whole-genome shotgun project has been deposited at DDBJ/EMBL/GenBank under the accession no. LAVB00000000. The version described in this paper is version LAVB01000000.
